# Single-Image Depth Inference Using Generative Adversarial Networks

**DOI:** 10.3390/s19071708

**Published:** 2019-04-10

**Authors:** Daniel Stanley Tan, Chih-Yuan Yao, Conrado Ruiz, Kai-Lung Hua

**Affiliations:** 1 Department of Computer Science and Information Engineering, National Taiwan University of Science and Technology, Taipei 10607, Taiwan; D10515805@mail.ntust.edu.tw (D.S.T.); cyuan.yao@csie.ntust.edu.tw (C.-Y.Y.); 2 Software Technology Department, De La Salle University, Manila 1004, Philippines; conrado.ruiz@dlsu.edu.ph; 3 Center for Cyber-Physical System Innovation, National Taiwan University of Science and Technology, Taipei 10607, Taiwan

**Keywords:** depth estimation, encoder-decoder networks, generative adversarial networks

## Abstract

Depth has been a valuable piece of information for perception tasks such as robot grasping, obstacle avoidance, and navigation, which are essential tasks for developing smart homes and smart cities. However, not all applications have the luxury of using depth sensors or multiple cameras to obtain depth information. In this paper, we tackle the problem of estimating the per-pixel depths from a single image. Inspired by the recent works on generative neural network models, we formulate the task of depth estimation as a generative task where we synthesize an image of the depth map from a single Red, Green, and Blue (RGB) input image. We propose a novel generative adversarial network that has an encoder-decoder type generator with residual transposed convolution blocks trained with an adversarial loss. Quantitative and qualitative experimental results demonstrate the effectiveness of our approach over several depth estimation works.

## 1. Introduction

Depth estimation is the task of inferring the distances of every point in a scene with respect to the camera. The main purpose is to represent the spatial structure of a scene. For an image, this translates to inferring the distance or depth value of every pixel. It is a crucial information for robot perception specially for performing tasks such as robot grasping [1], obstacle avoidance [2], and navigation [3], which are essential tasks for building smart homes and smart cities.

The inclusion of depth information has also been shown to enhance the performance of a wide range of models. As such, many research have developed creative ways of using the additional depth information to address various computer vision and image processing problems. Huang et al. [4] utilized depth information for error concealment in video transmission. Yang et al. [5,6] leveraged on depth video to better track and measure patients’ chest, abdominal movements, and heart rate over time. Shotton et al. [7], Shen et al. [8], and Lopes et al. [9] developed human pose recognition and correction models using depth images. Hu et al. [10], Devanne et al. [11], and Liu et al. [12] leveraged on the depth information to improve human movement and action recognition. Wang et al. [13], Yang et al. [14], and Husain et al. [15] showed that incorporating depth also improved semantic segmentation tasks.

Estimating depth is naturally done in stereo where another image is available with a slightly different perspective [16]. After all, humans rely heavily on the disparity of the images formed by our two eyes to perceive depth. Consequently, most prior works utilized stereo based techniques and reduced the problem into finding point correspondences and disparity matching [17,18,19,20]. However, requiring two cameras can be limiting, which is why researchers became creative and came up with various techniques to vary scene and shooting conditions in order to obtain two or more slightly different images of the same scene. Ge et al. [21] used a sliding camera to induce motion for depth estimation. Li et al. [22] and Zhang et al. [23] relied on light fields from varying illuminations. Yang, Liu, and Tang [24] exploited the symmetry in reflections in order to infer depths from water reflections.

Having only a single Red, Green, and Blue (RGB) image to estimate depth is an example of an ill-posed problem. This is due to the inherent ambiguity of measuring distances in an image. A large object that is farther away may appear exactly the same as a significantly smaller object that is positioned much closer to the camera. This ambiguity arises from the inevitable loss of information when we project a 3D scene into a 2D image. Even as humans, we rely heavily on the binocular disparity produced by the difference in the images seen by our left and right eyes. We have difficulties perceiving depth using only one eye. But we are able to leverage on our familiarity with the typical sizes for objects and combine them with visual cues for depth such as the shading, perspective, and occlusion [25,26,27,28]. Phan et al. [29] developed a more interactive approach by relying on user labeling to synthesize depth maps.

**Figure 1:**
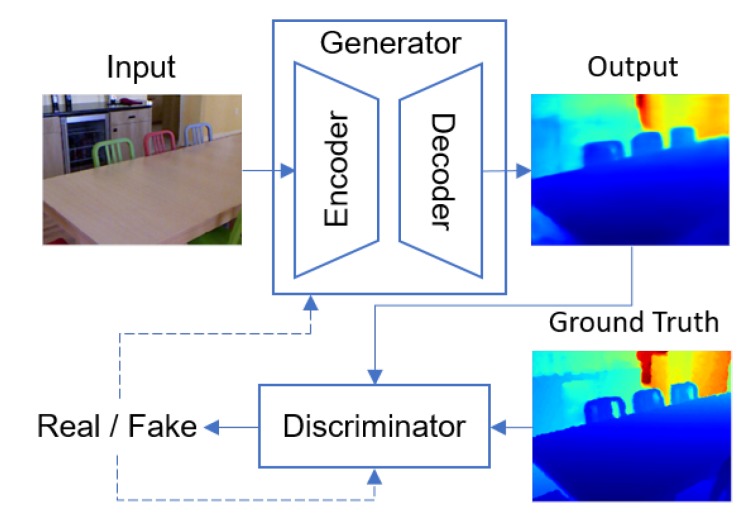
An overview of our encoder-decoder framework. The dashed line refers to the feedback coming from the output of the discriminator.

With the assumption that most objects that we encounter daily does not vary drastically in shape and size, we can rule out many of the unrealistic realizations of a scene and make it possible to infer depth from a single image. Eigen et al. [30] pioneered the use of simple neural networks for depth estimation. They later on extended their work to encorporate a more complex architecture [31]. Recently, generative neural network models trained with adversarial losses have been successful in tackling similarly ill-posed problems that require the model to imagine and synthesize realistic images given limited information [32,33,34]. Inspired by their works, we formulate the problem of monocular depth estimation as a generative image synthesis problem where our model receives a single RGB image as an input and produces a synthesized single-channel image as an output where the pixel values represent log distances from the camera. Figure 1 shows an overview of our approach. We propose a novel encoder-decoder type architecture with residual transposed convolution blocks and train it in a generative adversarial framework by adding a discriminator that differentiates between the real depth maps and the generated depth maps. The output of the discriminator helps the generator produce more realistic depth maps.

## 2. Related Works

Prior to the deep learning era, traditional approaches for depth estimation using only a single image relied on the relationship between geometric cues and depth to manually engineer features that can infer the geometry of the scene. Hoiem et al. [35] estimates a 3D contextual frame of the scene by inferring a coarse orientation of large surfaces as either facing left, right, or toward the camera, as well as whether the surface is part of the ground or sky. Ladicky et al. [36] discretized the depth values and phrased the problem into a pixel-wise depth classification. Karsch et al. [37], on the other hand, used SIFT flow warping and optical flow to formulate an optimization problem over the data, spatial smoothness and a database prior to generate the most likely depth map. Konrad et al. [38] leveraged on the correlation between photometric properties and 3D content of a scene to learn depths from a database of image-depth pairs. Their idea assumes images that are photometrically similar would have similar 3D structure and therefore depth. They used a simple *k*-nearest neighbor to search for photmetrically similar images and applied median filter and cross-bilateral filter to the *k* nearest depth maps.

To add more structure to the learning problem, Saxena et al. [39] employed probabilistic graphical models particularly Markov random fields to capture multi-scale local and global features in order to estimate depths of individual pixels. Liu, Gould, and Koller [40] extended this approach by first grouping pixels into a larger block called superpixels and before constructing a Markov random field over the superpixels to enforce global constraints on the connectivity and co-planarity of neighboring superpixels. Liu, Salzmann, and He [41] on the other hand, used discrete-continuous conditional random fields on the superpixels where the continuous variables correspond to the depth values of the superpixels and the discrete variables encode additional information on the neighborhood structure of the superpixels.

With the recent breakthroughs of deep learning, it is not surprising that the state-of-the-art models use more expressive models such as Convolutional Neural Networks (CNN). Eigen et al. [30] pioneered the use of using CNN’s for depth estimation. They used a two stage framework where they first used an AlexNet based architecture to produce a coarse depth map of a scene at a global level followed by a another CNN that makes local refinements to the depth map. The same authors later made a deeper and more discriminative model following the VGG architecture [31] to capture multi-scale representations of the depth map. They also showed that the exact same network architecture could also be used for surface normal estimation and semantic segmentation.

Roy and Todorovic [42] further extended the idea of having a multi-stage/multi-component framework and proposed a Neural Regression Forest which combines random forests and convolutional neural networks. The idea is to create an ensamble of CNNs in a tree like fashion where every node is tied to a small local network. Chakrabarti, Shao, and Shakhnarovich [43] had a similar idea where they trained multiple smaller neural networks as a predictor of the local geometric properties across overlapping patches. These patches are then harmonized using a globalization procedure. Wang et al. [13] and Liu et al. [44] also trained on smaller image patches but instead of a rectangular patch, they trained on superpixels. They also added a more explicit constraint on the structure by incorporating hierarchical conditional random fields to combine the patch-wise predictions and project it down to the pixel level.

In this paper, we propose a generative approach using an encoder-decoder type network to synthesize the depth maps given a single RGB image. We train this with an adversarial loss to encourage the synthesized images to look more real or in our case look similar to the ground truth depth maps.

## 3. Our Proposed Method

### 3.1. Problem Formulation

Formally, the problem of single image depth estimation is to learn a non-linear function F:I→D that maps an RGB image input I∈I in the image space to its corresponding real-valued depth map D∈D in the depth space. Given *N* image-depth pairs ${\left\{\left(I_{i},D_{i}\right)\right\}}_{i=1}^{N}$ as training data, we approximate this function F using a deep convolutional neural network. Instead of directly regressing to the ground-truth depth values D, we design our model to estimate the depths in the log space (logD) instead. This has been shown to produce better and more stable results [30,31].

We chose an encoder-decoder architecture because of its properties and recent success stories. Bousmalis et al. [45] showed that we can design a network that can learn domain-invariant features by adding training a domain classifier from the encoded representation. Hoffman et al. [46] and Wulmeier et al. [47] extended this for classification and semantic segmentation tasks. Tran, Yin, and Liu [33] leveraged on an encoder decoder architecture to learn how to separate the representations of pose and identity of a person. These ideas may potentially be harnessed for depth estimation and this paper initiates the first step by designing an encoder-decoder architecture suitable for depth estimation.

### 3.2. Network Architecture

Generative adversarial networks (GAN) have changed the landscape of generative modeling in the past few years. It has shown remarkable performance in representation learning [33,48] and enabled transforming data from one domain to another [32,34,49]. The fundamental idea behind their success is the inclusion of the discriminator. It transformed the learning problem into a game, more specifically a two-player minimax game, where the optimal solution is a Nash equilibrium.

The framework of a generative adversarial network starts with a generator *G* and a discriminator *D*. The role of the discriminator is to learn how to tell apart real images from synthetic images. In our case, the real images would correspond to the ground truth depth maps D and the synthetic images would be the generated depth maps $\hat{D}=G\left(I\right)$. The generator *G* synthesizes depth maps from an input I and tries to make it as realistic looking as possible in order to trick the discriminator *D* into classifying G(I) as real. This is represented as a min-max optimization in the form shown in Equation (1), where p(D) and p(I) represent the distributions of the depth map D and input image I. In the formulation of these networks, the generator *G* can cheat in the sense that it has access to the gradients of the discriminator *D* and therefore has some form of instruction as to how to improve itself. This enables the generator to learn how to produce realistic looking images.

(1) $$\begin{array}{cc} \min_{G}\max_{D}L_{\mathrm{gan}} & =E_{D∼p(D)}\left[\log D\left(D\right)\right]\\ & +E_{I∼p(I)}\left[\log\left(1-D\left(G\left(I\right)\right)\right)\right] \end{array}$$

**Figure 2:**
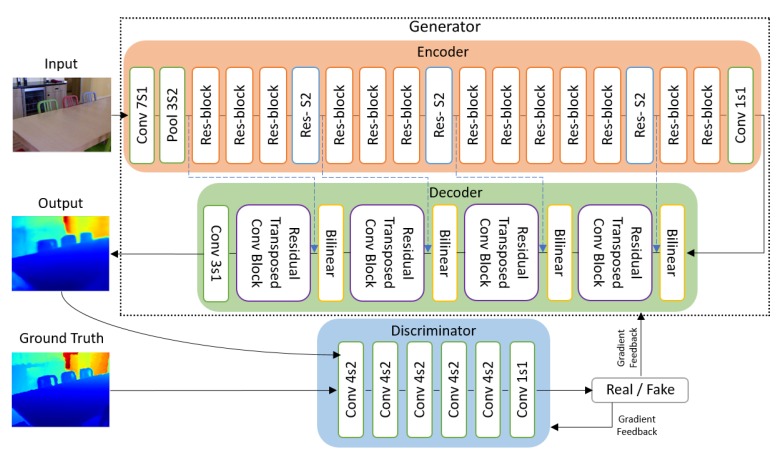
This figure shows a diagram of our proposed network architecture. The details of the residual transposed convolution block is discussed in Section 3.2.2 and further illustrated in Figure 3b.

Figure 2 shows an overview of our network architecture. For the generator, we adopt an encoder-decoder structure where we have an encoder $G_{\mathrm{enc}}$ that projects the input image into a lower dimensional representation and a decoder $G_{\mathrm{dec}}$ that reconstructs the depth map based on only the output of the encoder. For the generator to be successful, the encoder needs to efficiently learn and extract features which are essential for reconstructing the depth map. We use a ResNet50 based architecture [50] for our encoder $G_{\mathrm{enc}}$ as it strikes a balance between the model capacity and size. It is a powerful feature extractor and has a much deeper architecture than the usual VGG16 and VGG19 [51] architectures but it has a significantly lower memory footprint due to the efficient downsampling and removal of fully connected layers. As for the decoder $G_{\mathrm{dec}}$, we propose to use bilinear upsampling layers followed by residual transposed convolution blocks. We found that adopting the idea of residuals and skip connection to work well for reconstructing the depth map. Further details are discussed in the succeeding subsections.

For our discriminator, we employed a PatchGAN architecture proposed by Isola et al. [32]. Previous discriminators were designed to be binary classifiers that outputs the probability of an input being real or fake. The PatchGAN looks at the structure of local image patches and classify each patch in an image as real or fake instead of classifying the whole image as real or fake. This eliminates the need for the fully connected layers which reduces the number of parameters to be learned and enables it to be applied to arbitrarily large images.

#### 3.2.1. Bilinear Upsampling Layer

Since the encoder projects our image into a lower dimensional representation, we need to perform upsampling in order to bring the image back to the size or dimension of interest. The usual way of upsampling the image representations is to use transposed convolutions (also called fractional convolutions or deconvolutions in some literature) with stride two or more. Unfortunately, transposed convolutions suffer from uneven overlaping of the kernel during an upsampling step [52]. As a consequence, we often see checkerboard artifacts in its output, as illustrated by [52], which is very common in many generative models. To avoid this, we need a different upsampling operation that is differentiable for the backpropagation to still work. It should also retain the information as much as possible. We employed a bilinear interpolation as a means to upsample the image representations. It is computationally cheap and does not introduce any additional parameters.

Another advantage of using bilinear interpolation over transposed convolution is that the resulting dimensions are no longer constrained by possible values from convolution arithmetic. We can directly upsample image representations directly to the desired dimensions. This makes it straightforward to include in any deep learning model as well as to add skip connections among the layers of the network. One drawback of replacing the transposed convolutions to bilinear interpolations though is that it does not have any learnable parameters nor the capability to learn the appropriate depth values given the features from the encoder. To address this, we added our modified residual transposed convolution block after the upsampling step.

#### 3.2.2. Residual Transposed Convolution Block

**Figure 3:**
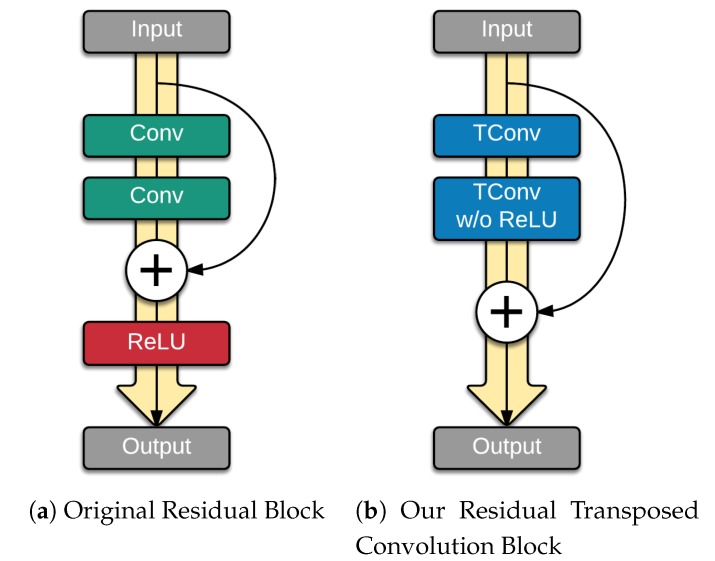
Difference between the original residual block and our modified residual block. (**a**) Original residual block as proposed by [50]. (**b**) Our proposed modification of the residual block.

With the success of the residual networks proposed by He et al. [50], we explored modeling the residuals for the decoder as well since it had superior performance in many tasks. The idea was to add skip connections where the gradients can skip and flow better throughout the network. We make two modifications to the residual block proposed by [50]. Inspired by [53], we removed the activation and batch normalization on the last convolution layer and output the sum of its result together with the input to the residual block. Figure 3 shows the difference between the original residual block and our modified residual block. The idea behind the modification is that actual residuals may have negative values, adding a ReLU activation before the output will restrict the values to only positives.

The other modification is to change the convolution layers into transposed convolutions (also referred to as deconvolutions or fractional convolutions). Note that in this case the transposed convolutions are not being used to upsample the image representation and therefore does not suffer from the checkerboard issue.

#### 3.2.3. Encoder-Decoder Skip Connections

Adding skip connections has been shown to significantly improve the training of very deep neural networks [50,53,54,55,56]. The skip connections act as information highways that enhance information propagation by adding shortcuts for the gradient to flow instead of restricting them to pass through multiple weight layers. The lower layers also get gradient signals faster through these shortcuts and thus reduce the vanishing gradient problem [55].

Inspired by the works of [32] and [56], we add skip connections from the layers in the encoder to the layers in the decoder, as illustrated by the dashed lines in Figure 2. In the problem that we are considering, the input and output seem very different. After all, the input is a three-channel RGB image with pixel intensities representing color and the output on the other hand is a single channel with pixel values representing distance. However, the input and the output share similar structure that makes the lower level features valuable in reconstructing the depth maps. By adding skip connections, it enables the encoder to pass information directly to the decoder. The decoder now has access to image representations as well as lower level features in the layers closer to the input image and thus makes it easier for the decoder to reconstruct depth maps that looks similar to the input image.

#### 3.2.4. Loss Function

To optimize min-max GAN objective shown in Equation (1), we split the objective function and independently optimize the generator network *G* and discriminator network *D* through alternating steps between the two. For the discriminator *D* updates, we can formulate its loss function $L_{\mathrm{gan}}^{D}$ using a standard binary cross entropy as shown in Equation (2). The first term accounts for the objective where we want to classify depth maps coming from the dataset as real, while the second term accounts for the objective where we want the depth maps produced by the generator *G* to be classified as fake. Note that while the objective function uses the results of the generator *G*, only the discriminator *D* is updated at this step.

(2) $$\begin{array}{cc} \min_{D}L_{\mathrm{gan}}^{D} & =-E_{D∼p(D)}\left[\log D\left(D\right)\right]\\ & -E_{I∼p(I)}\left[\log\left(1-D\left(G\left(I\right)\right)\right)\right] \end{array}$$

For the generator *G* updates, we can ignore the first term of Equation (1) since it does not rely on the generator *G* and will not matter in the optimization procedure. The GAN objective function of the generator $L_{\mathrm{gan}}^{G}$ is defined in Equation (3). We can interpret this objective function as the maximizing the probability of the generated depth maps as being real. Similar to the discriminator updates, we only update the generator network in this step.

(3) $$\begin{array}{c} L_{\mathrm{gan}}^{G}=-E_{I∼p(I)}\left[\log D\left(G\left(I\right)\right)\right] \end{array}$$

During the alternating updates of the generator *G* and the discriminator *D*, the discriminator *D* learns to differentiate the ground truth depth maps from the generated depth maps, while the generator *G* learns to produce more realistic depth maps that can fool the discriminator. However, the GAN objective alone is not enough for the generator *G* to produce accurate depth maps. Therefore, we include another objective $L_{\mathrm{task}}^{G}$ that encourages the generator *G* to minimize the squared euclidean norm of the differences between the ground truth D and the predicted depths $\hat{D}$ (as shown in Equation (4)), which is the actual task that we are interested in. The final objective of the generator is now defined in Equation (5), where $\lambda_{\mathrm{task}}$ and $\lambda_{\mathrm{gan}}$ control the relative importance of the two objectives $L_{\mathrm{task}}^{G}$ and $L_{\mathrm{gan}}^{G}$ respectively.

(4) $$L_{\mathrm{task}}^{G}={∥D-G\left(I\right)∥}^{2}$$

(5) $$\min_{G}L_{\mathrm{final}}^{G}=\lambda_{\mathrm{task}}L_{\mathrm{task}}^{G}+\lambda_{\mathrm{gan}}L_{\mathrm{gan}}^{G}$$

## 4. Experiments

In this section, we discuss the evaluation results of our model qualitatively and quantitatively. We also perform ablation experiments to demonstrate the effectiveness of our proposed design.

### 4.1. Dataset

The standard benchmark dataset for indoor depth estimation is the NYU Depth v2 dataset [57]. The raw dataset offers 464 video sequences of indoor scenes amounting to 407,024 frames taken from diverse indoor environments such as bedrooms, bathrooms, and living rooms, as well as offices, libraries, and furniture stores. The depth information was captured using the depth camera from the Microsoft Kinect. They also provide an official train and test split with a total of 795 training images coming from 249 scenes and 654 for testing images coming from the remaining 215 scenes. We used the official test set to evaluate our models in order to be consistent and comparable with previous works.

From the scenes available on the training set, we randomly sampled 20,000 unique image frames from the raw video sequences. This is one order of magnitude lower than the amount of unique frames that Eigen et al. [30,31] used. We then downsample the size of the images to half (240 × 320) from their original size of 480 × 640.

Due to factors, such as noise, shadows, and low albedo surfaces, some portions of the depth maps have missing values in them. Following the recommendation of [57], we employ the colorization scheme of Levin et al. [58] to fill in the missing values. We kept the dimensions of the ground truth depth maps in the test set untouched to keep the integrity of the evaluations.

### 4.2. Data Augmentation

One of the difficulties in working with indoor scenes is that there are potentially an unbounded number of variations that a scene can take on. Bedrooms, for example, may look drastically different from one house to another, and this is just one type of scene. Since we have limited data, performing data augmentations are essential to accommodate some of these variations and artificially increase the data. This helps the model to be more robust to slight changes.

Following previous works, we scaled both the input image and its corresponding depth map with a scaling factor *s* followed by a center crop to the desired input size of 240 × 320. This induces an effect of zooming into the scene. However, for the depth maps, this operation does not preserve the world-space geometry of the original scene [30]. This can be corrected by dividing the depth values by the corresponding scaling factor *s*. We scaled the images at s=1.2 and s=1.5 in additional to the original scaling. Aside from scaling, we also flipped the images horizontally. All these were performed offline and saved as one big training set.

We also perform some augmentations on the fly. To accommodate for noise and small changes induced by the environment and image capturing process, randomly shift the brightness and gamma values on the image. We also randomly perturb the colors of the RGB image slightly. Each of these augmentations were assigned a probability of 50% of executing during training.

### 4.3. Implementation Details

Training GANs have been notoriously known for being unstable. One of the problems is that in the beginning the discriminator does not yet know how to differentiate real from fake, and thus gives poor instructions towards the generator. The opposite can also happen where either the generator or the discriminator is performing too good for the other to catch up [59]. This is why some people often come up with various schedules on training GANs. In our work we train the generator and discriminator alternately with one update step each.

We minimize the loss function defined in Equation (5) for the generator and Equation (2) for the discriminator. We set $\lambda_{\mathrm{task}}=10$ and $\lambda_{\mathrm{gan}}=1$. We used the YellowFin optimizer [60], which automatically tunes the learning rate and momentum of a stochastic gradient descent optimizer. We initialized it with a learning rate of 0.001 and used a batch size of 8. We also linearly scaled the intensities of the RGB images to the range [0,1] as a form of normalization for our inputs.

### 4.4. Evaluation

We compare against several baseline methods on single image depth estimation to benchmark our proposed method. A summary of the baseline methods are as follows:

- Karsch et al. [37] proposed a non-parametric approach where they first find candidate images that are most similar to the input image from a prior database containing the images and their corresponding depth maps. Next, they warp the candidate images and depth maps using sift and optical flow features to align it with the input image. Lastly, they used the warped candidates to formulate an optimization problem that minimizes three terms: a data term that measures the distance of the predicted depth maps to the warped candidates, a spatial smoothness term that encourages the depth values to have small intensity gradients, and a database prior term that incorporates the assumptions of the database.
- Ladicky et al. [36] discretized the depth values and phrased the problem of depth estimation into pixel-wise depth classification. They train a multi-class boosted classifier [61] from extracted features such as textons [62], SIFT [63], local quantized ternary patterns [64], and self similarirty features [65] for each pixel.
- M. Liu et al. [41] first clusters the pixels of the image into a set of superpixels. They then used discrete-continuous conditional random fields on the superpixels to predict the depth values, where the continuous variables correspond to the depth values of the superpixels and the discrete variables encode additional information on the neighborhood structure of the superpixels.
- F. Liu et al. [44] predicts the depth maps of superpixels using a convolutional neural network. They then use a conditional random field to impose smoothness and consistency among neighboring superpixels.
- Wang et al. [13] used two convolutional neural networks to model both depth and semantic labels on a global and local scale. To combine the predictions, they used a hierarchical conditional random field.
- Eigen et al. [30] used an AlexNet based architecture to produce a coarse depth map of the scene at a global level followed by another convolutional neural network that makes local refinements to the predicted depth map.
- Eigen and Fergus [31] proposed a multi-scale and multi-task convolutional neural network that jointly predicts the depth maps, surface normals, and semantic labels. The idea is that the knowledge learned by each of the tasks can be shared, which can further improve the performance of the model as compared to learning each of the tasks independently.
- Roy and Todorovic [42] proposed a multi-stage and multi-component framework to predict depth maps using neural regression forests, which combines random forests with convolutional neural networks. They create an ensemble of networks in a tree-like fashion where every node is tied to a small local network. The predicted depth values are then averaged over the trees.
- Chakrabarti et al. [43] used Gaussian mixture models (GMM) to model the distribution of depth derivatives at different orientation and scales across small overlapping patches. The mixture weights of the GMM are inferred using convolutional neural networks.

We follow prior works and use standard metrics to evaluate the predicted depth maps $\hat{D}$. The evaluation metrics are listed below, where *M* refers to the size of the test set, $D_{i}$ denotes the ground truth depth values, and ${\hat{D}}_{i}$ denotes the predicted depth values:

- (rel) Mean Relative Error $\frac{1}{M}\sum_{i=1}^{M}\left|D_{i}-{\hat{D}}_{i}\right|/D_{i}$
- (rmse) Root Mean Squared Error $\sqrt{\frac{1}{M}\sum_{i=1}^{M}\left|\right|D_{i}-{\hat{D}}_{i}{\left|\right|}^{2}}$
- (log10) Log10 error $\frac{1}{M}\sum_{i=1}^{M}\left|\log_{10}D_{i}-\log_{10}{\hat{D}}_{i}\right|$
- (δ) Thresholded Accuracy: % of $D_{i}$ s.t. $\left[\max\left(\frac{D_{i}}{{\hat{D}}_{i}},\frac{{\hat{D}}_{i}}{D_{i}}\right)=\delta \right]<$ threshold, where threshold $\in \{1.25,1.25^{2},1.25^{3}\}$

The first three metrics (rel, rmse, log10) are error metrics meaning the lower the values are, the better the performance. The thresholded accuracy on the other hand is an accuracy measure wherein we compute the percentage of predicted depth values ${\hat{D}}_{i}$ that have a ratio less than a specified threshold with respect to the ground truth values $D_{i}$. This means a higher value would mean better performance.

Table 1 shows the performances of the different methods. Aside from the relative error, our approach outperforms prior state-of-the-art methods. To analyze why our network has a higher relative error even though it achieved a lower root mean squared error, let us consider two simple cases. Let $\hat{p}$ denote one pixel on the predicted depth map and *p* be the corresponding ground truth depth value for that pixel. Suppose in the first case $\hat{p}=9.0$ and p=8.5, and in the second case $\hat{p}=2.0$ and p=1.5. Both of these cases will have the same absolute difference of 0.5, and therefore have the same root mean squared error. However, their relative errors will be 0.33 and 0.06 respectively, showing that relative errors have more weight for lower pixel values. The $\ell_{2}$ loss, on the other hand, penalizes larger differences more, particularly on the differences larger than one. Since this is the objective that our network directly optimizes for, it indirectly results in our network prioritizing the farthest portions as these are more likely to have larger differences especially during the early stages of training.

**Figure 4:**
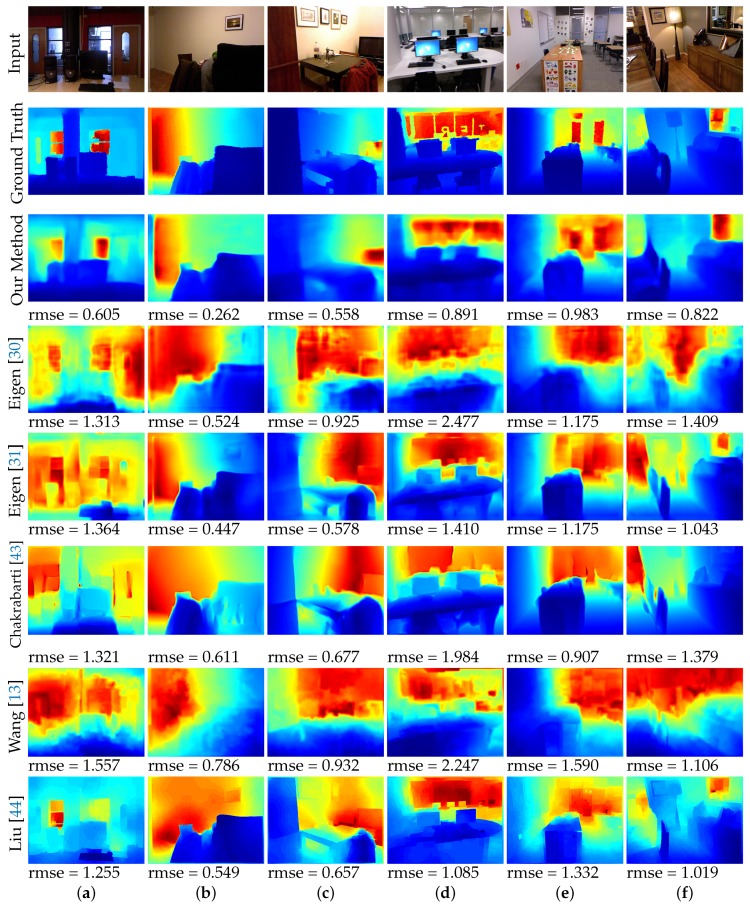
Visual comparison of the output of our model with the several state-of-the-art baselines. All the results in this figure were reproduced based on the respective author’s publicly available results and codes.

We also qualitatively evaluate our method through visual assessment. Figure 4 shows examples of the predicted depth maps. We only compared against the methods with publicly available results or code. Visually, the output of our model looks like a silhouette of the input image. This implies that our model is able to capture the low frequency components well. The smoothened edges can be attributed to the $\ell_{2}$ loss objective. Our model is also able to capture some of the structural details which is an improvement as compared to [30,31] which had a similar objective function as ours.

### 4.5. Failure Cases

**Figure 5:**
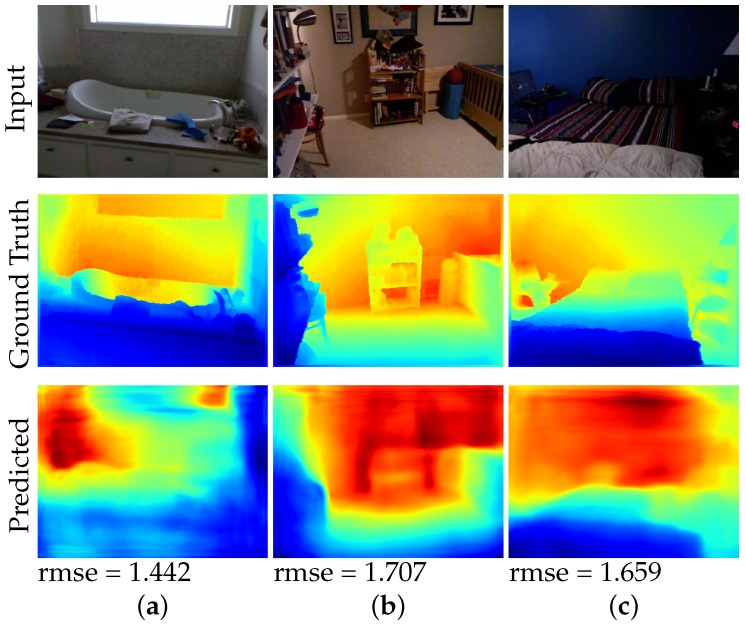
Example failure cases of our method.

While our method produces promising results as compared to several baseline methods, there is still room for improvement. Figure 5 shows some example failure cases of our method. We observe that our method tend to overestimate the depth on images that are captured close to the wall and facing the wall. We hypothesize that this is due to most of the images in the dataset are images of rooms or hallways that have a wide range of depth values. This may bias our network in also predicting a wide range of depth values for all images, which leads overestimating the depth of images that are directly facing a wall.

## 5. Conclusions

This paper presents a generative approach for the problem of depth estimation using only a single image. We designed an encoder-decoder architecture with our introduced residual transposed convolution blocks for our decoder. We also added skip connections from the encoder to the decoder that helps propagate information by adding a more direct channel for message passing between the encoder and the decoder. We evaluated our method both quantitatively and qualitatively, demonstrating that our approach achieves excellent approximation of the depth information and outperforms several baseline methods for single image depth estimation.

While depth estimation can be estimated better using stereo images it requires two cameras, which not only costs more but also uses more computational resources since we have to process two images and compute for correspondences. The technology to estimate depth from a single image can reduce these requirements and can potentially be used in settings where there are resource constraints. This technology is also beneficial for applications where we do not have access to stereo images. These cases can arise in applications such as detecting and recognizing objects, estimating the room layout, converting 2D images to 3D, or reconstructing the 3D scene from a 2D image. 

## Figures and Tables

**Table 1:** This table shows the comparison of our method with the several state-of-the-art baselines. The values are adopted from their respective papers. The bold fonts represent the best performing result. The dashes are missing values on the particular evaluation metric not reported by the authors in their paper.

	Error (Lower Is Better)			Accuracy (Higher Is Better)		
	rel	log10	rmse	δ<1.25	$\delta <1.25^{2}$	$\delta <1.25^{3}$
Karsch et al. [37]	0.349	0.134	1.214	0.447	0.745	0.897
Ladicky et al. [36]	-	-	-	0.542	0.829	0.941
M. Liu et al. [41]	0.335	0.127	1.060	-	-	-
F. Liu et al. [44]	0.230	0.095	0.824	0.614	0.883	0.975
Wang et al. [13]	0.220	0.094	0.745	0.605	0.890	0.970
Eigen et al. [30]	0.215	-	0.907	0.611	0.887	0.971
Roy and Todorovic [42]	0.187	0.078	0.744	-	-	-
Eigen and Fergus [31]	0.158	-	0.641	0.769	0.950	**0.988**
Chakrabarti et al. [43]	**0.149**	-	0.620	0.806	0.958	0.987
**Ours**	0.176	**0.074**	**0.597**	**0.867**	**0.965**	**0.988**

## Abbreviations

The following abbreviations are used in this manuscript:

RGB	Red, Green, and Blue
3D	3 Dimensional
2D	2 Dimensional
SIFT	Scale-invariant feature transform
CNN	Convolutional Neural Networks
GAN	Generative Adversarial Network
rel	Mean Relative Error
rmse	Root Mean Squared Error
log10	Log10 error
